# Pharmacotherapy of Psychotic Disorders in Türkiye: Insights From a National Prescription Database

**DOI:** 10.1002/brb3.70712

**Published:** 2025-07-26

**Authors:** Sena Türkeş, Aybeniz Civan Kahve, Taner Çin, Esra Şafak Yılmaz, Hakkı Gürsöz, Süreyya Barun

**Affiliations:** ^1^ Department of Medical Pharmacology, Faculty of Medicine Gazi University Ankara Türkiye; ^2^ Department of Psychiatry, Faculty of Medicine Gazi University Ankara Türkiye; ^3^ Department of Medical Informatics Sağlık Bilimleri University Gülhane Faculty of Medicine Ankara Türkiye; ^4^ Institue of Public Health Ankara Yıldırım Beyazıt University Ankara Türkiye

**Keywords:** antipsychotic agents, prescriptions, psychotic disorders, schizophrenia

## Abstract

**Objective:**

Psychotic disorders, affecting 3–7 per 1000 individuals, significantly impair mental functioning. Although antipsychotic medications represent the mainstay of treatment, prescribing practices can vary based on patient characteristics and across different countries. Currently, national epidemiological data on psychotropic prescribing patterns in Türkiye are lacking, and understanding these variations is crucial for informing treatment guidelines and optimizing patient care.

**Methods:**

Data from the Turkish Prescription Information System (PIS) for the year 2019 were analyzed to evaluate antipsychotic prescribing patterns for psychotic disorders. Included were prescriptions for schizophrenia, other specified psychotic disorders not attributable to a substance or known physiological condition, and unspecified psychosis with the same exclusion criteria.

**Results:**

Second‐generation antipsychotics (SGAs) constituted 80% of all prescriptions, making them the most frequently prescribed class in Türkiye. This finding is consistent with other national studies. Quetiapine was the most frequently prescribed SGA overall, although prescribing patterns varied by age group. Although SGAs were the most commonly prescribed class, typical antipsychotics or first‐generation antipsychotics (FGAs) remained in use, particularly haloperidol in patients aged ≥80 years.

**Conclusions:**

This national study demonstrates the widespread use of SGAs in Türkiye, particularly quetiapine, while also highlighting the continued use of FGAs, especially haloperidol, in older adults. These findings highlight the need for further research on age‐specific prescribing practices and the development of tailored treatment guidelines. Furthermore, international comparisons of prescribing patterns should be conducted to identify potential variations and explore their underlying causes.

## Graphical abstract

This 2019 national study evaluated antipsychotic prescriptions for patients ≥18 with schizophrenia or psychotic disorders across age, medication, region, and diagnosis. SGAs, especially quetiapine, were most prescribed, whereas sertindole, zuclopenthixol, haloperidol, chlorpromazine, and ziprasidone were least frequent. SGA use peaked in patients ≥80, where haloperidol was the most common FGA.

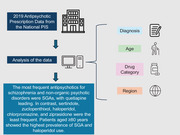

## 1 Introduction

Psychotic disorders, characterized by disturbances in perception of reality, emotional responses, cognition, and behavior, present with heterogeneous symptom profiles and variable clinical courses (Boland et al. 2022). These disorders significantly impact individuals’ functioning and quality of life.

Schizophrenia, a prototypic psychotic disorder, typically emerges between the ages of 15 and 25 in men and 20 and 30 in women (Häfner 2019). The incidence of psychotic disorders ranges from 3 to 7 per 1000 individuals, reflecting methodological variations across studies (Moreno‐Küstner et al. 2018; Saha et al. 2005). Pharmacotherapy, primarily with antipsychotic medications, is the cornerstone of treatment, aiming to reduce symptoms, minimize functional impairment, and improve social adaptation (Herz et al. 2000).

Antipsychotics are broadly classified as first‐generation (typical) and second‐generation (atypical) agents. Although both classes effectively reduce positive psychotic symptoms, second‐generation antipsychotics (SGAs) are generally preferred due to their reduced risk of extrapyramidal side effects, leading to improved adherence (Freudenreich 2020). Although the American Psychiatric Association recommends person‐centered care for schizophrenia (American Psychiatric Association 2020), the heterogeneity of clinical trial designs and limited comparative data preclude definitive recommendations for specific antipsychotic selection, with the exception of clozapine's established superior efficacy in treatment‐resistant cases.

The Psychiatric Association of Türkiye also recommends SGAs as first‐line treatment for psychotic disorders, acknowledging the need for individualized treatment approaches (Soygür et al. 2007). Although some studies have explored psychotropic use in smaller samples of Turkish patients, national epidemiological data on prescribing patterns remain scarce. Therefore, this study aims to evaluate all psychotropic prescriptions for psychotic disorders in Türkiye over a 1‐year period. This research is expected to contribute to the development of evidence‐based treatment guidelines and provide a basis for international comparisons of prescribing practices.

## 2 Materials and Methods

### 2.1 Data Source and Study Population

This study analyzed data from the Turkish Medicines and Medical Devices Agency's Prescription Information System (PIS). Ethical approval was obtained from the Gazi University Ethics Committee. The PIS contains electronic prescription data submitted by healthcare providers across Türkiye.

This study included prescriptions issued by family physicians in 2019 for adult patients (≥18 years) diagnosed with schizophrenia (ICD‐10 code: F20) or nonorganic psychotic disorder (ICD‐10 codes: F28 and F29, combined as “nonorganic psychotic disorder” due to clinical similarities). The reason for using data only from 2019 is that the agency only shared data from this year with the study team. In Türkiye, patients with mental health complaints are initially evaluated by a psychiatrist, who may then authorize family physicians to prescribe psychotropic medications for long‐term management through specialized reports. The analyzed data, therefore, reflect prescriptions issued within this framework of shared care. A list of included medications is provided in Table 1. Prescription rates were compared across Nomenclature of Territorial Units for Statistics (NUTS) regions.

**TABLE 1:** Anatomical therapeutic chemical (ATC) codes of the drugs included in the study.

Generic name	ATC code
Chlorpromazine	N05AA01
Fluphenazine	N05AB02
Trifluoperazine	N05AB06
Haloperidol	N05AD01
Sertindole	N05AE03
Ziprasidone	N05AE04
Flupentixol	N05AF01
Zuclopenthixol	N05AF05
Pimozide	N05AG02
Clozapine	N05AH02
Olanzapine	N05AH03
Quetiapine	N05AH04
Sulpiride	N05AL01
Amisulpride	N05AL05
Lithium	N05AN01
Risperidone	N05AX08
Aripiprazole	N05AX12
Paliperidone	N05AX13
Diazepam	N05BA01
Lorazepam	N05BA06
Alprazolam	N05BA12
Hydroxyzine	N05BB01
Buspirone	N05BE01
Zopiclone	N05CF01
Ramelteon	N05CH02

### 2.2 Statistical Analyses

Statistical analyses were performed using SPSS version 25.0 (IBM Corp., Armonk, NY, USA). Categorical variables were compared using the chi‐square test, whereas the Kruskal–Wallis test was used to compare means across more than two groups. Statistical significance was set at *p* < 0.05.

## 3 Results

**FIGURE 1:**
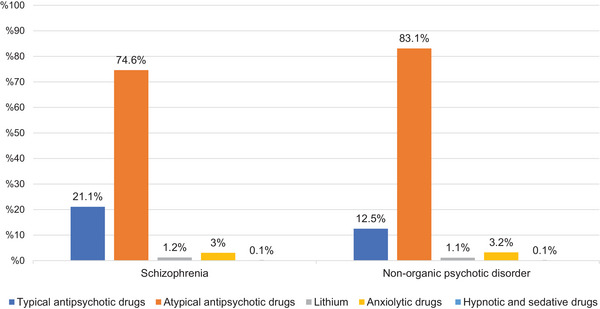
Percentage distribution of antipsychotics by diagnostic group.

A total of 457,098 prescriptions were analyzed, including 149,337 (32.7%) for schizophrenia (F20) and 307,761 (67.3%) for nonorganic psychotic disorders (F28 and F29). Antipsychotics constituted the majority of prescriptions in both groups: 142,881 (95.7%) in the schizophrenia group and 294,277 (95.6%) in the nonorganic psychotic disorder group. Atypical antipsychotics were the most frequently prescribed medication class in both diagnostic groups (*p* < 0.05), followed by typical antipsychotics, anxiolytics, and lithium (*p* < 0.05). The distribution of antipsychotic medications within each diagnostic group is shown in Figure 1.

**FIGURE 2:**
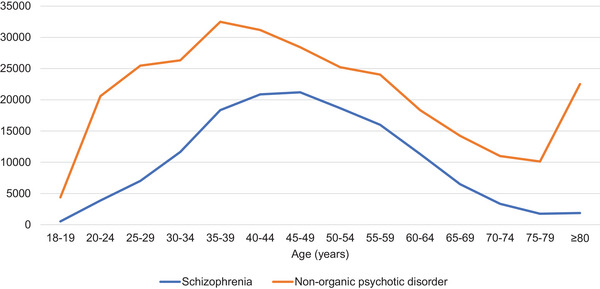
Antipsychotic prescriptions by age.

Antipsychotic prescribing patterns varied by age group and diagnosis (Figure 2). For schizophrenia (F20), prescription numbers peaked between 40 and 49 years of age, with 20,861 (12.8%) and 21,199 (14.8%) prescriptions for the 40–44 and 45–49 age groups, respectively. In contrast, prescriptions for nonorganic psychotic disorders (F28 and F29) peaked earlier, between 35 and 44 years of age (32,490 (11%) prescriptions for 35–39 years and 31,174 (10.6%) for 40–44 years), generally declining thereafter, with a subsequent increase observed in the ≥80 age group.

**FIGURE 3:**
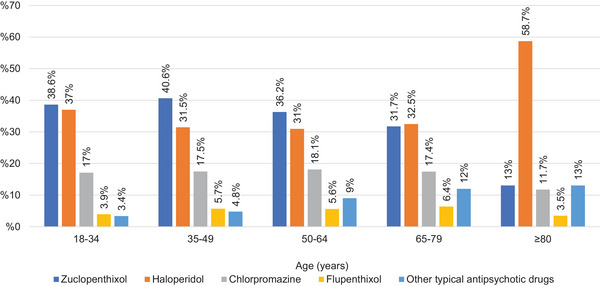
Percentages of typical antipsychotics prescribed for schizophrenia.

**FIGURE 4:**
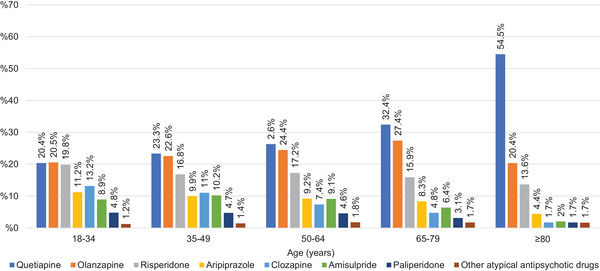
Percentages of atypical antipsychotics prescribed for schizophrenia.

A total of 149,337 psychotropic prescriptions were issued for schizophrenia (F20). Atypical antipsychotics were prescribed more frequently than typical antipsychotics across all age groups (*p* < 0.05). The most frequently prescribed antipsychotics were quetiapine (*n* = 27,735, 19.4%), olanzapine (*n* = 25,765, 18.0%), and risperidone (*n* = 19,323, 13.5%) (Table 2). Typical antipsychotic prescriptions peaked in the 50–64 age group, whereas atypical antipsychotic prescriptions peaked in the ≥80 age group. The proportion of atypical antipsychotic prescriptions generally decreased until the age of 65 and subsequently increased. Zuclopenthixol and haloperidol were the most frequently prescribed typical antipsychotics in the 18–34 and 65–79 age groups (*p* < 0.05), with no significant difference between their prescription numbers in these groups. Zuclopenthixol was the most frequently prescribed typical antipsychotic in the 35–49 and 50–64 age groups (*p* < 0.05). In the ≥80 age group, haloperidol was the most frequently prescribed typical antipsychotic, accounting for 58.7% of prescriptions (*p* < 0.05). Quetiapine, olanzapine, and risperidone were the most frequently prescribed atypical antipsychotics across all age groups. The distribution of typical and atypical antipsychotic prescriptions by age group is presented in Figures 3 and 4.

**TABLE 2:** The numbers and percentages of drugs prescribed for schizophrenia and nonorganic psychotic disorder.

Schızophrenıa			Nonorganıc psychotıc dısorder		
Active substance	Number of prescriptions	%	Active substance	Number of prescriptions	%
Quetiapine	27,735	19.41	Quetiapine	84,977	28.9
Olanzapine	25,765	18.03	Olanzapine	59,166	20.1
Risperidone	19,323	13.52	Risperidone	51,542	17.5
Zuclopenthixol	11,896	8.33	Aripiprazole	27,626	9.39
Aripiprazole	10,829	7.58	Haloperidol	13,947	4.74
Clozapine	10,758	7.53	Amisulpride	13,364	4.54
Amisulpride	10,261	7.18	Zuclopenthixol	12,634	4.29
Haloperidol	10,127	7.09	Paliperidone	8303	2.82
Chlorpromazine	5551	3.89	Chlorpromazine	6807	2.31
Paliperidone	5024	3.52	Clozapine	5957	2.02
Flupenthixol	1736	1.21	Sulpiride	3901	1.33
Sulpiride	1111	0.78	Trifluoperazine	1850	0.63
Fluphenazine	1072	0.75	Flupenthixol	1797	0.61
Trifluoperazine	688	0.48	Ziprasidone	911	0.31
Ziprasidone	553	0.39	Fluphenazine	822	0.28
Pimozide	423	0.30	Pimozide	635	0.22
Sertindole	29	0.02	Sertindole	38	0.01

**FIGURE 5:**
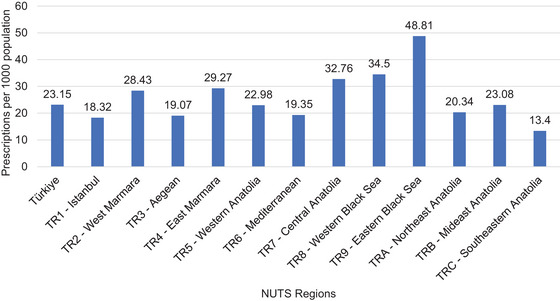
Prescriptions for schizophrenia by NUTS regions.

Regional variations in antipsychotic prescribing for schizophrenia were observed (Figure 5). Prescription rates per 10,000 population were highest in the Eastern Black Sea region and lowest in Southeastern Anatolia.

**FIGURE 6:**
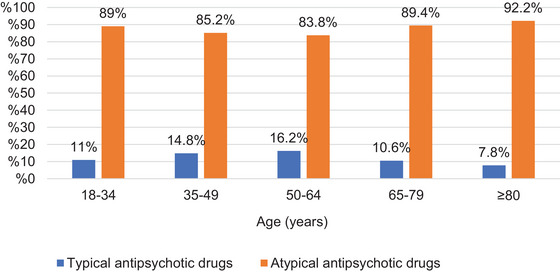
Percentages of antipsychotics for nonorganic psychotic disorder.

A total of 307,761 psychotropic prescriptions were issued for nonorganic psychotic disorders. Similar to schizophrenia prescriptions, atypical antipsychotics were prescribed more frequently than typical antipsychotics across all age groups (*p* < 0.05). The most frequently prescribed antipsychotics were quetiapine (*n* = 84,977; 28.9%), olanzapine (*n* = 59,166; 20.1%), and risperidone (*n* = 51,542; 17.5%) (Table 2). The proportion of typical antipsychotic prescriptions was highest in the 50–64 age group, whereas the proportion of atypical antipsychotic prescriptions was highest in the ≥80 age group (*p* < 0.05). The distribution of prescriptions by age group is shown in Figure 6.

**FIGURE 7:**
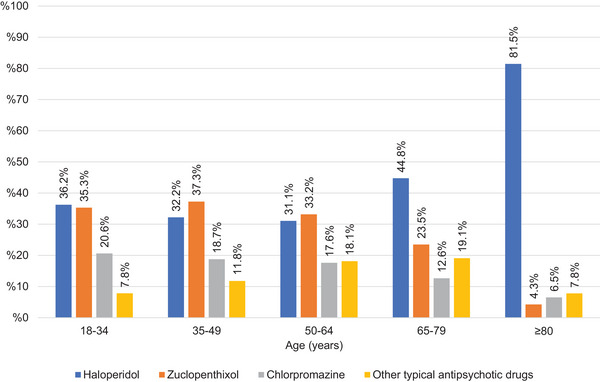
Percentages of typical antipsychotics for nonorganic psychotic disorder.

Within the typical antipsychotic class, zuclopenthixol and haloperidol were the most frequently prescribed medications in the 18–34 age group (*p* < 0.05). Zuclopenthixol was the most frequently prescribed typical antipsychotic in the 35–49 and 50–64 age groups (*p* < 0.05). Haloperidol was the most frequently prescribed typical antipsychotic in the 65–79 and ≥80 age groups (*p* < 0.05). The distribution of typical antipsychotic prescriptions by age group is presented in Figure 7.

**FIGURE 8:**
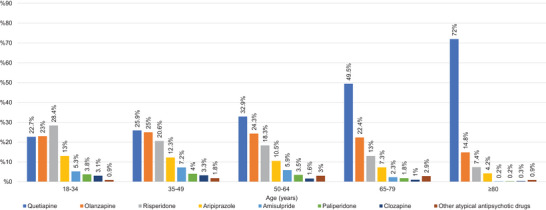
Percentages of atypical antipsychotics for nonorganic psychotic disorder.

Risperidone was the most frequently prescribed atypical antipsychotic in the 18–34 age group (*p* < 0.05). Quetiapine, olanzapine, and risperidone were the most frequently prescribed atypical antipsychotics in the 35–49, 50–64, 65–79, and ≥80 age groups, respectively (*p* < 0.05). The distribution of atypical antipsychotic prescriptions by age group is presented in Figure 8.

**FIGURE 9:**
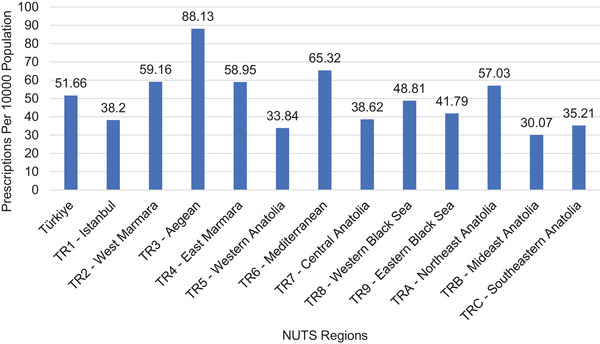
Prescriptions for nonorganic psychotic disorder by NUTS regions.

Regional variations in antipsychotic prescribing for nonorganic psychotic disorders were observed (Figure 9). Prescription rates per 10,000 population were highest in the Aegean region and lowest in the Mideast Anatolia region.

**FIGURE 10:**
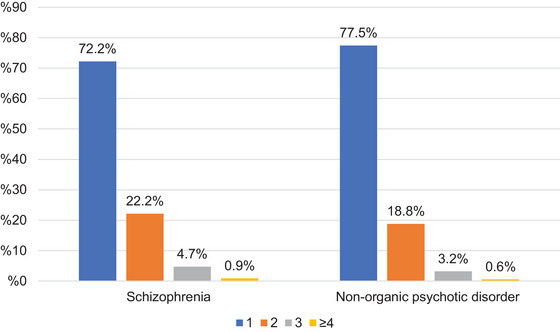
Number of medications per prescription.

Figure 10 shows the distribution of N05A medication prescribing patterns (monotherapy vs. polypharmacy) by diagnostic group. Monotherapy was the most frequent pattern in both schizophrenia and nonorganic psychotic disorders (*p* < 0.05), accounting for 72.2% and 77.5% of prescriptions, respectively.

## 4 Discussion

This study revealed that atypical antipsychotics (SGAs) were the predominant class of antipsychotics prescribed across all age groups in Türkiye, consistent with international trends (Hálfdánarson et al. 2017). However, notable differences emerged in prescribing patterns for schizophrenia (F20) compared to nonorganic psychotic disorders (F28 and F29). Although antipsychotic prescriptions for schizophrenia peaked in the 40–49 age group, aligning with reported peak onset ages (Charlson et al. 2018), prescriptions for nonorganic psychotic disorders peaked earlier, in the 35–39 age group. This earlier peak may reflect diagnostic ambiguity in initial presentations or reluctance to assign a schizophrenia diagnosis due to associated stigma (Aker et al. 2002; Taskin et al. 2002). The observed increase in nonorganic psychotic disorder prescriptions in the ≥80 age group may also reflect diagnostic challenges in differentiating psychosis from age‐related cognitive decline (Nilsson et al. 2018).

Quetiapine, risperidone, and olanzapine were the most frequently prescribed antipsychotics overall, consistent with findings from other countries (Hálfdánarson et al. 2017; Højlund et al. 2019) while showing minor variations compared to data from other countries (Hashimoto et al. 2021; Huskamp et al. 2016; Marston et al. 2014). Our findings are generally consistent with studies examining prescribing patterns in smaller patient populations in Türkiye. One study analyzing outpatient prescriptions at a single center found a higher rate of atypical antipsychotic prescribing compared to typical antipsychotics, and the most frequently prescribed atypical antipsychotics were similar to those in our study (Atik et al. 2008). Another study examining prescriptions at a different center also found atypical antipsychotics to be most frequent; however, zuclopenthixol depot ranked third (Yildiz and Cerit 2004). The frequent use of zuclopenthixol, a typical antipsychotic, may be attributed to its long‐acting injectable formulation (Verdoux et al. 2017; Nasrallah 2007). Haloperidol use increased in older patients, likely due to its favorable side effect profile, ease of titration, and availability in various formulations (Ozbolt et al. 2008). Although quetiapine use also increased with age, possibly due to its perceived safety profile, risperidone use tended to decrease, potentially due to concerns about cardiovascular side effects in older adults.

The least frequently prescribed antipsychotics in limited sample studies conducted in Türkiye have been reported as sertindole, zuclopenthixol, haloperidol, chlorpromazine, and ziprasidone. Aligning with these observations, our study also found these agents to be among the least frequently prescribed, which may be attributed to their adverse effect profiles leading to their consideration as lower priority options by clinicians in routine practice (Atik et al. 2008; Yazici et al. 2017).

Regional variations in prescribing rates were observed, with higher rates in regions with greater physician density and healthcare access, as reported by the Ministry of Health (Berrak Bora Başara et al. n.d.). This finding contradicts a study that observed higher antipsychotic prescriptions in the most deprived regions (Marston et al. 2014). Furthermore, other studies also indicate regional differences in prescribing. These studies suggest that these differences may be attributed to factors such as sociocultural and historical contexts, medication costs and health insurance coverage, varying prescribing habits among doctors in different regions, and different preferences for the adoption of new medications. The reasons for regional disparities in Türkiye could be elucidated by more comprehensive studies (Donohue et al. 2016; Park et al. 2018). This suggests potential disparities in access to specialized mental healthcare across Türkiye.

Although antipsychotic monotherapy was the most common prescribing pattern, approximately 25% of prescriptions involved polypharmacy, which is lower than findings from other studies (Hashimoto et al. 2021; Yang et al. 2018). Unlike our findings, a study previously discussed that examined patients at community counseling centers in Türkiye reported a higher rate of polypharmacy compared to monotherapy, suggesting disease severity as a predictor. This difference from our results may stem from the possibility that patients in that study, attending such centers, had treatment‐resistant schizophrenia (Yazici et al. 2017). Further research is needed to explore the rationale for combined antipsychotic use and to promote evidence‐based prescribing practices.

This study, on the basis of national prescription data, provides a comprehensive overview of antipsychotic prescribing patterns in Türkiye. However, it is limited by its reliance on diagnostic codes from prescription data, which may be subject to inaccuracies. Future pharmacoepidemiologic studies incorporating patient‐level data are needed to further explore these findings and to inform clinical practice and policy.

## Author Contributions

**Sena Türkeş**: conceptualization, investigation, writing–original draft. **Aybeniz Civan Kahve**: conceptualization, supervision, writing–review and editing. **Taner Çin**: visualization, writing–review and editing, validation. **Esra şafak Yılmaz**: formal analysis, resources, visualization. **Hakkı Gürsöz**: investigation, resources, supervision. **Süreyya Barun**: conceptualization, project administration, supervision.

## Ethics Statement

The present study was conducted subsequent to the ethical approval of the Gazi University Ethics Committee.

## Conflicts of Interest

The authors declare no conflicts of interest.

## Declaration of Generative AI and AI‐Assisted Technologies in the Writing Process

During the preparation of this study, the author used DeepL Write by DeepL SE to rephrase the sentences. After using this service, the author reviewed and edited the content as needed and takes full responsibility for the content of the publication.

## Peer Review

The peer review history for this article is available at https://publons.com/publon/10.1002/brb3.70712

## Data Availability

The data from this research are available upon reasonable request.
